# Ten-Year Results of a Single-Center Trial Investigating Heart Rate Control with Ivabradine or Metoprolol Succinate in Patients After Heart Transplantation

**DOI:** 10.3390/jcdd12080297

**Published:** 2025-08-01

**Authors:** Fabrice F. Darche, Alexandra C. Alt, Rasmus Rivinius, Matthias Helmschrott, Philipp Ehlermann, Norbert Frey, Ann-Kathrin Rahm

**Affiliations:** 1 Department of Cardiology, Angiology and Pneumology, Heidelberg University Hospital, 69120 Heidelberg, Germany; 2 Heidelberg Center for Heart Rhythm Disorders (HCR), Heidelberg University Hospital, 69120 Heidelberg, Germany

**Keywords:** heart transplantation, ivabradine, metoprolol succinate, mortality, survival, sinus tachycardia

## Abstract

**Aims:** Sinus tachycardia after heart transplantation (HTX) due to cardiac graft denervation is associated with reduced post-transplant survival and requires adequate treatment. We analyzed the long-term effects of heart rate control with ivabradine or metoprolol succinate in HTX recipients. **Methods:** This observational retrospective single-center study analyzed the ten-year results of 110 patients receiving ivabradine (*n* = 54) or metoprolol succinate (*n* = 56) after HTX. Analysis included comparison of demographics, medications, heart rates, blood pressure values, echocardiographic features, cardiac catheterization data, cardiac biomarkers, and post-transplant survival including causes of death. **Results:** Both groups showed no significant differences concerning demographics or medications (except for ivabradine and metoprolol succinate). At 10-year follow-up, HTX recipients with ivabradine showed a significantly lower heart rate (72.7 ± 8.5 bpm) compared to baseline (88.8 ± 7.6 bpm; *p* < 0.001) and to metoprolol succinate (80.1 ± 8.1 bpm; *p* < 0.001), a significantly lower NT-proBNP level (588.4 ± 461.4 pg/mL) compared to baseline (3849.7 ± 1960.0 pg/mL; *p* < 0.001) and to metoprolol succinate (1229.0 ± 1098.6 pg/mL; *p* = 0.005), a significantly lower overall mortality (20.4% versus 46.4%; *p* = 0.004), and mortality due to graft failure (1.9% versus 21.4%; *p* = 0.001). Multivariate analysis showed a significantly decreased risk of death within 10 years after HTX in patients with post-transplant use of ivabradine (HR 0.374, CI 0.182–0.770; *p* = 0.008). **Conclusions:** In this single-center trial, patients with ivabradine revealed a significantly more pronounced heart rate reduction, a lower NT-proBNP level, and a superior 10-year survival after HTX.

## 1. Introduction

Elevated resting heart rates lead to increased myocardial oxygen demand, diastolic dysfunction and shortened diastolic filling resulting in decreased stroke volume and reduced myocardial perfusion [1,2,3,4,5,6,7]. Accordingly, increased morbidity and mortality have been linked to elevated resting heart rates in the general population as well as in patients with heart failure [8,9,10,11].

As a result of cardiac denervation after heart transplantation (HTX), the autonomous control of heart rate regulation by the vagus nerve is diminished, and HTX recipients usually suffer from inadequate sinus tachycardia [1,2,3,4,5,6,7]. Although a higher resting heart rate can help stabilize the patient in the early post-transplant stage by increasing cardiac output, in the long-term, higher resting heart rates in HTX recipients have been related to reduced post-transplant survival [6,7,12].

Given these circumstances, a specific and selective therapeutic agent with a minimum of possible side effects for the treatment of sinus tachycardia in HTX recipients is needed to achieve physiological resting heart rates [1,2,3,4,5,6,7]. Standard pharmacological management of tachycardia includes the use of beta blockers or non-dihydropyridine calcium channel blockers [1,2,3,4,5,6,7,13]. However, both drug classes are non-specific inhibitors of pacemaker activity and can cause side effects such as atrioventricular block, negative inotropy, hypotension, bronchospasm, depression, fatigue, and sexual dysfunction [1,2,3,4,5,6,7,14].

In contrast to beta blockers or calcium channel blockers, ivabradine is a specific and selective inhibitor of pacemaker activity [1,2,3,4,5,6,7,14,15,16,17,18]. Ivabradine inhibits the so-called funny current (If), also known as “pacemaker current”, generated by hyperpolarization-activated cyclic nucleotide-gated (HCN) channels in pacemaker cells [1,2,3,4,5,6,7,14,15,16,17,18]. Although ivabradine is considered to exhibit an overall good safety profile, its heart rate-lowering properties can cause bradycardia and respective QT interval prolongation as the QT interval depends on heart rate [19,20,21,22]. Long-term effects of ivabradine on QT and QTc interval in HTX recipients are unknown, especially relating to the co-administration of potentially QT-prolonging drugs such as tacrolimus [23]. We therefore investigated the long-term effects (ten-year results) of heart rate control with ivabradine or metoprolol succinate in HTX recipients focusing on concomitant medications, heart rates, QT/QTc intervals, echocardiographic features, cardiac catheterization data, cardiac biomarkers, and survival after HTX.

## 2. Patients and Methods

### 2.1. Patients

We carried out this study in compliance with the ethical principles set forth in the Declaration of Helsinki. The institutional review board (IRB) of Heidelberg University, Heidelberg, Germany, issued ethics approval number: S-286/2015, Version 1.2, 28 July 2020. Written informed consent was obtained from patients for enrollment in the Heidelberg HTX Registry and for the clinical and scientific use of their data. The ethics approval does not require additional consent for this observational retrospective single-center as we used only routine clinical data [6,7,24,25,26].

We screened the medical data of all adult patients (≥18 years) for continuous post-transplant use of ivabradine or metoprolol succinate (in the following context referred to as metoprolol) who underwent HTX at Heidelberg Heart Center, Heidelberg, Germany, between 2006 and 2015. HTX recipients were excluded if they were only temporarily treated with ivabradine or metoprolol, received a combination of ivabradine and metoprolol, or were treated with additional antiarrhythmic drugs (amiodarone, digoxin/digitoxin, other beta blockers, or non-dihydropyridine calcium channel blockers) [6,7]. Patients who had undergone repeat HTX were also excluded [6,7].

### 2.2. Follow-Up

Follow-up of HTX recipients adhered to the routine clinical protocol of Heidelberg Heart Center [6,7,24,25,26]. As part of HTX surgery, patients routinely received a temporary pacemaker system consisting of an external pacing box and two epicardial pacing leads that were placed on the right atrium and ventricle during HTX surgery. The epicardial pacing leads remained in situ for about 10 days after HTX [26]. If clinically indicated, HTX recipients could be over-paced with this temporary pacemaker. As soon as HTX recipients were clinically stable, they were discharged and followed up at the HTX outpatient clinic. During the first visit at the HTX outpatient clinic, HTX recipients presented for baseline follow-up, which included routine assessment of resting heart rate and initialization of heart rate control with ivabradine or metoprolol succinate. HTX recipients were neither preselected nor randomized for treatment with ivabradine versus metoprolol to manage post-transplant heart rate. Individual physician practice and patient preference influenced the prescription of either drug, reflecting real-world data [6,7]. As the use of ivabradine in HTX recipients is still off-label, HTX recipients were informed beforehand about effects, adverse effects, contraindications, and the off-label use of ivabradine [6,7].

At baseline after HTX, the initial standard daily ivabradine dose was 10 mg (2 × 5.0 mg), and the initial standard daily metoprolol succinate dose was 100 mg (2 × 50 mg or 1 × 100 mg), which are both adequate starting doses. Slight variations in this scheme occurred due to clinical reasons (individual physician practice and/or patient preference). Adaption of the doses of ivabradine and metoprolol succinate were managed during follow-up visits, aiming for a durable resting heart rate ≤ 80 bpm. HTX recipients were seen monthly at the HTX outpatient clinic during the first six post-transplant months, then bimonthly until the end of the first year after HTX, and approximately three to four times per year thereafter. From five years after HTX onward, routine follow-up visits were reduced to once or twice annually (with additional visits as clinically needed) [6,7,24,25,26].

Standard follow-up assessments consisted of medical history, physical examination, systolic and diastolic blood pressure measurement, laboratory tests including immunosuppressive drug monitoring, resting 12-lead ECG, echocardiography, endomyocardial biopsy, annual chest X-ray, as well as annual 24 h Holter monitor [6,7,24,25,26].

Complete 10-year follow-up data after HTX were available for all 110 HTX recipients; no HTX recipient was lost to follow-up. As part of post-transplant care, patients were regularly assessed for medication use, tolerability, and potential complications to support adherence [6,7,24,25,26].

### 2.3. Post-Transplant Medications

Post-transplant pharmacotherapy, including immunosuppressive regimens, followed the center’s standard [6,7,24,25,26]. Perioperatively, HTX recipients received anti-thymocyte globulin-based immunosuppression induction therapy. Most HTX recipients in this study received tacrolimus and mycophenolic acid as immunosuppressive drug regime, given the fact that cyclosporine A was subsequently replaced by tacrolimus as the primary immunosuppressive drug from 2006 onward. Patients also received steroids (prednisolone), which were gradually tapered and, when clinically feasible, discontinued six months after HTX [6,7,24,25,26].

### 2.4. Statistical Analysis

Data analysis was performed with MedCalc (Version 23.2.1, MedCalc Software Ltd., Ostend, Belgium) and presented as mean ± standard deviation (SD) or as count (*n*) with percentage (%). Mean difference (MD) with 95% confidence interval (CI) was used for measures of association. Depending on data type and study question, statistical testing employed—as appropriate in each case—Student’s *t*-test, Mann–Whitney U-test, analysis of variance (ANOVA), Kruskal–Wallis test, chi-squared test, or Fisher’s exact test. Heart rates [measured by beats per minute (bpm)] between HTX recipients with ivabradine or metoprolol were annually assessed by resting 12-lead ECG over a period of ten years after HTX and graphically displayed by a box-plot diagram. Post-transplant survival between groups was graphically compared with Kaplan–Meier curves and the log-rank test. Figures were created with CorelDRAW Graphics Suite 2025 (Version 26.0.0.101; Corel Corporation, Ottawa, ON, Canada). A *p*-value of <0.050 denoted statistical significance [6,7,24,25,26].

We performed large-scale univariate analyses to search for intergroup differences between HTX recipients with ivabradine or metoprolol. Analyzed variables included recipient data, recipient previous open-heart surgery, recipient principal diagnosis for HTX, donor data, transplant sex mismatch, perioperative data, immunosuppressive drug therapy, post-transplant concomitant medications, resting ECGs, 24 h-Holter monitors, QT/QTc intervals, blood pressure values, echocardiographic features, cardiac catheterization data, and cardiac biomarkers after HTX [6,7,24,25,26,27].

Causes of death within ten years after HTX were categorized into the following groups: graft failure, acute rejection, infection/sepsis, malignancy, and thromboembolic event/bleeding. Analysis of 10-year post-transplant mortality between HTX recipients with ivabradine or metoprolol further included a multivariate analysis (Cox regression model) with the following eight clinically relevant parameters: recipient age, recipient body mass index (BMI), recipient estimated glomerular filtration rate (eGFR), donor age, donor BMI, transplant sex mismatch, total ischemic time, and administration of ivabradine after HTX. We did not include additional variables in this multivariate analysis for 10-year mortality after HTX to avoid biased regression coefficients and to ensure a stable number of events (deceased patients) per analyzed variable [6,7,24,25,26].

## 3. Results

### 3.1. Demographics and Post-Transplant Medications

After applying the exclusion criteria, a total of 110 HTX recipients were selected, including 54 patients with ivabradine after HTX (54 of 110 [49.1%]) and 56 patients with metoprolol after HTX (56 of 110 [50.9%]).

In terms of demographics, we found no statistically significant differences between both groups regarding recipient data, recipient previous open-heart surgery, recipient principal diagnosis for HTX, donor data, transplant sex mismatch, or perioperative data (all *p* ≥ 0.050). Demographic and clinical characteristics at baseline are presented in Table 1.

Analysis of post-transplant medications including the immunosuppressive drug regimen revealed no statistically significant differences between both groups (all *p* ≥ 0.05) except for the administration of ivabradine or metoprolol. Post-transplant medications at baseline, including the immunosuppressive drug regimen, are given in Table 2.

### 3.2. Drug Dosage and Side Effects

At baseline after HTX, HTX recipients with ivabradine were administered a mean daily dose of 9.8 mg ± 2.9 mg ranging from 5.0 mg to 15.0 mg, and HTX recipients with metoprolol were administered a mean daily dose of 98.0 mg ± 44.4 mg ranging from 47.5 mg to 190.0 mg. At 5-year follow-up after HTX, the mean daily ivabradine dose was 10.7 mg ± 3.6 mg ranging from 5.0 mg to 15.0 mg, and the mean daily metoprolol dose was 112.7 mg ± 51.0 mg ranging from 47.5 mg to 190.0 mg. At 10-year follow-up after HTX, the mean daily ivabradine dose was 10.8 mg ± 3.4 mg ranging from 5.0 mg to 15.0 mg, and the mean daily metoprolol dose was 111.6 mg ± 51.1 mg ranging from 47.5 mg to 190.0 mg.

Patients generally tolerated ivabradine well. Mild and temporary side effects were infrequent, with phosphenes reported transiently in three individuals (5.6%) during the initial treatment period. No HTX recipient with ivabradine reported symptomatic bradycardia (0.0%), while six HTX recipients with metoprolol reported symptomatic bradycardia with heart rates < 60 bpm (10.7%; *p* = 0.013). Only one HTX recipient with ivabradine reported intermittent dizziness (1.9%), whereas eight HTX recipients with metoprolol reported intermittent dizziness (14.3%; *p* = 0.017). No HTX recipient with ivabradine reported fatigue (0.0%), while five HTX recipients with metoprolol reported fatigue (8.9%; *p* = 0.025).

### 3.3. Post-Transplant Heart Rates

**Figure 1:**
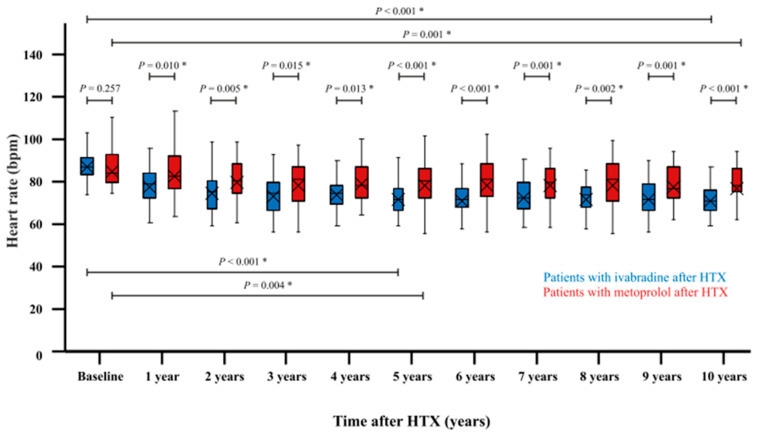
The 10-year course of average heart rates (resting ECG) in patients with ivabradine or metoprolol after HTX. There is no statistically significant difference in average heart rates (resting ECG) between HTX recipients starting treatment with ivabradine or metoprolol (*p* = 0.257). At 5-year follow-up after HTX, patients with ivabradine show a statistically significant lower average heart rate (resting ECG) in comparison to baseline after HTX (*p* < 0.001) and to patients with metoprolol at 5-year follow-up (*p* < 0.001). At 10-year follow-up after HTX, patients with ivabradine continue to have a statistically significant lower average heart rate (resting ECG) in comparison to baseline after HTX (*p* < 0.001) and to patients with metoprolol at 10-year follow-up (*p* < 0.001). BPM = beats per minute; HTX = heart transplantation; and * = statistically significant (*p* < 0.050).

At baseline after HTX, there was no statistically significant difference in average heart rates (resting ECG) between HTX recipients starting treatment with ivabradine or metoprolol (ivabradine at baseline: 88.8 ± 7.6 bpm versus metoprolol at baseline: 86.9 ± 9.5 bpm, *p* = 0.257). At 5-year follow-up after HTX, patients with ivabradine had a statistically significant lower average heart rate (resting ECG) in comparison to baseline after HTX (ivabradine at 5-year follow-up: 73.4 ± 9.0 bpm versus ivabradine at baseline: 88.8 ± 7.6 bpm, *p* < 0.001) and to HTX recipients with metoprolol (ivabradine at 5-year follow-up: 73.4 ± 9.0 bpm versus metoprolol at 5-year follow-up: 80.7 ± 10.3 bpm, *p* < 0.001). At 10-year follow-up after HTX, patients with ivabradine continued to have a statistically significant lower average heart rate (resting ECG) in comparison to baseline after HTX (ivabradine at 10-year follow-up: 72.7 ± 8.5 bpm versus ivabradine at baseline: 88.8 ± 7.6 bpm, *p* < 0.001) and to HTX recipients with metoprolol (ivabradine at 10-year follow-up: 72.7 ± 8.5 bpm versus metoprolol at 10-year follow-up: 80.1 ± 8.1 bpm, *p* < 0.001). Figure 1 illustrates the 10-year trajectory of average heart rates (resting ECG) in HTX recipients treated with ivabradine versus metoprolol.

Analysis of average heart rates (24 h-Holter monitor) at baseline showed no statistically significant difference between HTX recipients starting treatment with ivabradine or metoprolol (ivabradine at baseline: 86.2 ± 9.8 bpm versus metoprolol at baseline: 85.6 ± 9.0 bpm, *p* = 0.756). At 5-year follow-up after HTX, patients with ivabradine had a statistically significant lower average heart rate (24 h-Holter monitor) in comparison to baseline after HTX (ivabradine at 5-year follow-up: 72.5 ± 7.1 bpm versus ivabradine at baseline: 86.2 ± 9.8 bpm, *p* < 0.001) and to patients with metoprolol (ivabradine at 5-year follow-up: 72.5 ± 7.1 bpm versus metoprolol at 5-year follow-up: 79.9 ± 8.1 bpm, *p* < 0.001). At 10-year follow-up after HTX, patients with ivabradine continued to have a statistically significant lower average heart rate (24 h-Holter monitor) in comparison to baseline after HTX (ivabradine at 10-year follow-up: 70.9 ± 7.0 bpm versus ivabradine at baseline: 86.2 ± 9.8 bpm, *p* < 0.001) and to patients with metoprolol (ivabradine at 10-year follow-up: 70.9 ± 7.0 bpm versus metoprolol at 10-year follow-up: 79.1 ± 8.4 bpm, *p* < 0.001). Average heart rates (24 h-Holter monitor) of patients with ivabradine or metoprolol after HTX are given in Table 3.

### 3.4. QT/QTc Intervals

No statistically significant differences in baseline QT/QTc intervals were observed between the two groups (QT: *p* = 0.337, respectively, QTc: *p* = 0.672). At 5-year follow-up after HTX, both groups showed a statistically significant longer QT interval compared to baseline (ivabradine at 5-year follow-up: 391.9 ± 23.4 ms versus ivabradine at baseline: 365.5 ± 21.1 ms, *p* < 0.001, respectively, metoprolol at 5-year follow-up: 383.5 ± 23.8 ms versus metoprolol at baseline: 369.4 ± 21.2 ms, *p* = 0.004). In contrast, there was no statistically significant change concerning QTc interval at 5-year follow-up after HTX compared to baseline (ivabradine: *p* = 0.560, respectively, metoprolol *p* = 0.165). QTc intervals at 5-year follow-up after HTX did not differ significantly between patients treated with ivabradine and those receiving metoprolol (*p* = 0.199). At 10-year follow-up after HTX, both groups continued to show a statistically significant longer QT interval compared to baseline (ivabradine at 10-year follow-up: 392.8 ± 26.4 ms versus ivabradine at baseline: 365.5 ± 21.1 ms, *p* < 0.001, respectively, metoprolol at 10-year follow-up: 389.9 ± 21.3 ms versus metoprolol at baseline: 369.4 ± 21.2 ms, *p* < 0.001). There was still no statistically significant change concerning QTc interval at 10-year follow-up after HTX compared to baseline (ivabradine: *p* = 0.341, respectively, metoprolol *p* = 0.148). Likewise, there was no statistically significant difference between patients with ivabradine or metoprolol concerning QTc interval at 10-year follow-up after HTX (*p* = 0.423). QT/QTc intervals of patients with ivabradine or metoprolol after HTX are presented in Table 3.

### 3.5. Blood Pressure Values

Assessment of blood pressure values showed no statistically significant differences between HTX recipients with ivabradine or metoprolol concerning systolic blood pressure or diastolic blood pressure at baseline (*p* = 0.821, respectively, *p* = 0.469), at 5-year follow-up after HTX (*p* = 0.782, respectively, *p* = 0.790), and at 10-year follow-up after HTX (*p* = 0.963, respectively, *p* = 0.932). Blood pressure values are shown in Table 3.

### 3.6. Post-Transplant Mortality and Causes of Death

**Figure 2:**
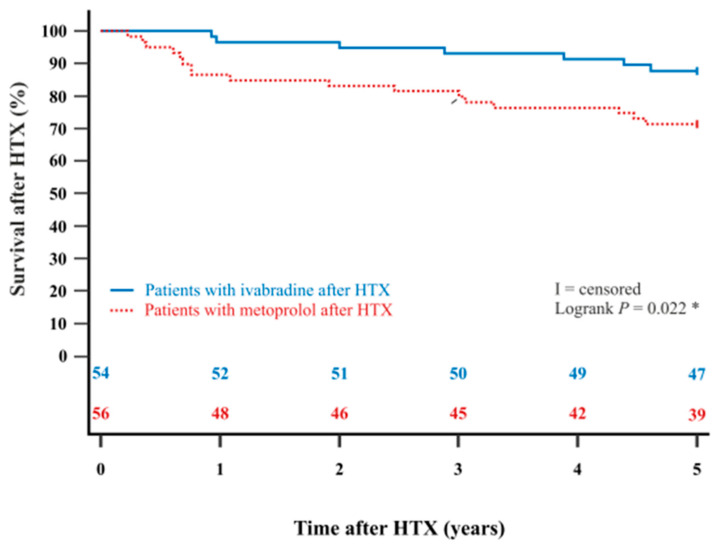
The 5-year survival after HTX between patients with ivabradine or metoprolol after HTX (Kaplan–Meier estimator). Patients with ivabradine after HTX have a significantly higher 5-year post-transplant survival than patients with metoprolol after HTX (*p* = 0.022). HTX = heart transplantation; * = statistically significant (*p* < 0.050).

**Figure 3:**
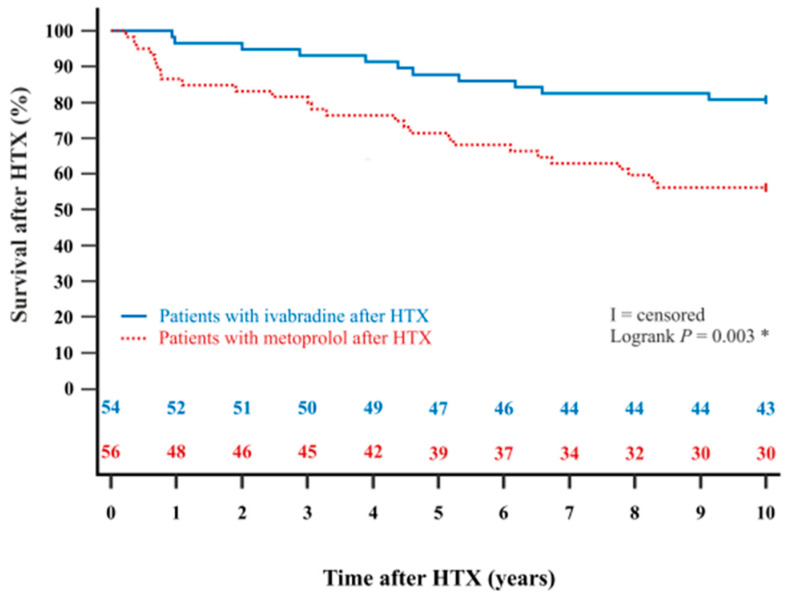
The 10-year survival after HTX between patients with ivabradine or metoprolol after HTX (Kaplan–Meier estimator). Patients with ivabradine after HTX have a significantly higher 10-year post-transplant survival than patients with metoprolol after HTX (*p* = 0.003). HTX = heart transplantation; * = statistically significant (*p* < 0.050).

The Kaplan–Meier estimator showed a significantly better 5-year post-transplant survival (*p* = 0.022) and 10-year post-transplant survival (*p* = 0.003) in HTX recipients with ivabradine in comparison to HTX recipients with metoprolol. Kaplan–Meier estimators are displayed in Figure 2 and Figure 3.

When examining causes of death, significantly fewer patients in the ivabradine group died from graft failure within five years after HTX (0.0% versus 16.0%, MD: 16.0%, CI: 6.4–25.6%, *p* = 0.002), and within ten years after HTX (1.9% versus 21.4%, MD: 19.5%, CI: 8.2–30.8%, *p* = 0.001) in comparison to the metoprolol group. No significant differences were observed between the two groups regarding acute rejection, infection/sepsis, malignancy, or thromboembolic events/bleeding at 5- or 10-year follow-up after HTX (all *p* ≥ 0.050). Table 4 presents the causes of death within five and ten years after HTX.

Multivariate analysis for 5-year mortality after HTX showed that patients in the ivabradine group had a significantly reduced risk of death within five years after HTX (HR: 0.388, 95% CI: 0.159–0.949, *p* = 0.038) and within ten years after HTX (HR: 0.374, 95% CI: 0.182–0.770, *p* = 0.008), whereas the other seven included variables (recipient age, recipient body mass index, recipient estimated glomerular filtration rate, donor age, donor body mass index, transplant sex mismatch, and total ischemic time) showed no statistically significant effect on 5-year or 10-year post-transplant mortality. The multivariate analysis for 5-year and 10-year mortality after HTX can be found in Table 5.

### 3.7. Post-Transplant Echocardiographic Features

Analysis of echocardiographic features at baseline showed no statistically significant differences between patients with ivabradine or metoprolol regarding left ventricular (LV) mass, LV mass index, left ventricular ejection fraction (LVEF), mitral annular plane systolic excursion (MAPSE), early diastolic mitral inflow peak velocity (E) to late diastolic mitral inflow peak velocity (A) ratio (E/A), E to early diastolic mitral annular velocity (e′) ratio (E/e′), deceleration time (DT) of E (DT-E), left atrial (LA) diameter, or systolic pulmonary artery pressure (PAP) (all *p* ≥ 0.050).

At 10-year follow-up after HTX, the ivabradine group showed a statistically significant reduction in LV mass (*p* = 0.004) and LV mass index (*p* = 0.001) towards normal values, whereas no such effect over time was observed in the metoprolol group in terms of LV mass (*p* = 0.874) or the LV mass index (*p* = 0.916). Furthermore, patients in the metoprolol group had a slight but significant decrease in LVEF (*p* < 0.001) and MAPSE (*p* < 0.001) at 10-year follow-up after HTX, while patients in the ivabradine group had no statistically significant change in LVEF (*p* = 0.314) or MAPSE (*p* = 0.515).

Assessment of diastolic parameters showed that patients in the metoprolol group experienced a significant decrease in E/A ratio (*p* = 0.005), an increase in E/e′ ratio (*p* = 0.002), and a stable DT-E (*p* = 0.117) at 10-year follow-up after HTX. In contrast, patients in the ivabradine group over time showed a stable E/A ratio (*p* = 0.169), a stable E/e′ ratio (*p* = 0.987), and a decrease in DT-E (*p* < 0.001). Additionally, patients with ivabradine showed an unaltered LA diameter (*p* = 0.076) and a lower systolic PAP (*p* = 0.003) at 10-year follow-up after HTX, whereas patients with metoprolol had an enlarged LA diameter (*p* < 0.001) and no decrease in systolic PAP (*p* = 0.915). Echocardiographic features after HTX are presented in Table 6.

### 3.8. Post-Transplant Cardiac Catheterization Data and Cardiac Biomarkers

Cardiac catheterization data showed no statistically significant differences between HTX recipients with ivabradine or metoprolol in coronary artery disease, coronary stenting, or high-sensitivity cardiac troponin T at baseline, at 5-year follow-up after HTX, or at 10-year follow-up after HTX (all *p* ≥ 0.050).

Patients in the ivabradine group showed a statistically significant lower left ventricular end-diastolic pressure (LVEDP) at 5-year follow-up after HTX (ivabradine group: 12.0 ± 3.7 mmHg versus metoprolol group: 17.1 ± 2.6 mmHg; *p* < 0.001) and at 10-year follow-up after HTX (ivabradine group: 10.4 ± 3.4 mmHg versus metoprolol group: 16.7 ± 2.5 mmHg; *p* < 0.001) as well as a statistically significant lower N-terminal prohormone of the brain natriuretic peptide (NT-proBNP) at 5-year follow-up after HTX (ivabradine group: 555.4 ± 541.9 pg/mL versus metoprolol group: 1021.0 ± 862.8 pg/mL; *p* = 0.004) and at 10-year follow-up after HTX (ivabradine group: 588.4 ± 461.4 pg/mL versus metoprolol group: 1229.0 ± 1098.6 pg/mL; *p* = 0.005). Cardiac catheterization data and cardiac biomarkers after HTX are presented in Table 7.

## 4. Discussion

### 4.1. Long-Term Management of Resting Heart Rates After Heart Transplantation

This study is the first to present 10-year data comparing heart rate control with ivabradine versus metoprolol in HTX recipients. We found that ivabradine was associated with a significantly better long-term heart rate reduction, which correlated with a lower NT-proBNP level, and an improved 10-year survival after HTX. Long-term management of resting heart rates after HTX is of high clinical relevance for HTX recipients as the current guidelines do not specifically address this issue and transplant cardiologists need to rely on the limited available studies for guidance [1,2,3,4,5,6,7]. Accordingly, center-specific practices have emerged over time but are not available to the public for review. The purpose of this study was therefore to provide insights into this complex topic.

In terms of resting heart rates, HTX recipients with ivabradine or metoprolol showed comparable resting heart rates at baseline after HTX. At 5-year and 10-year follow-up after HTX, the ivabradine group had significantly lower resting heart rates compared to both their baseline and the metoprolol group. The ivabradine group sustained a significantly lower resting heart rate than the metoprolol group over the entire 10-year period, indicating superior heart rate control with ivabradine. Comparable findings on the effectiveness of ivabradine in reducing heart rate among HTX recipients over shorter observation periods have been reported by Boeken and colleagues [28] as well as by Dos Santos and colleagues [29].

Variations in resting heart rate outcomes between HTX recipients with ivabradine or metoprolol could partially stem from differences in daily drug dosages. At baseline after HTX, the mean daily ivabradine dose was about 10 mg (2 × 5.0 mg), and the mean daily metoprolol dose was about 100 mg (2 × 50 mg or 1 × 100 mg), which are both adequate starting doses. Although both groups were titrated upwards over time, neither group reached the maximum doses (2 × 7.5 mg for ivabradine; 2 × 100 mg or 1 × 200 mg for metoprolol) due to reports of temporary asymptomatic bradycardia (heart rate < 60 bpm) during patient self-monitoring [4,5,6,7]. Therefore, while greater heart rate reductions may have been possible with higher doses in both groups, the similarity in dose titration suggests that dosage differences had minimal impact on overall heart rate outcomes.

Additionally, analysis of 24 h-Holter monitors at 5-year and 10-year follow-up after HTX showed significantly lower average heart rates in the ivabradine group compared to both their baseline and the metoprolol group, while there was no significant difference in average heart rates between both groups at baseline. These findings underscore the superior efficacy of ivabradine in reducing heart rate in denervated cardiac grafts following HTX, where autonomic control is lost. Ivabradine exerts its effect by directly inhibiting sinoatrial node activity, thereby lowering heart rate independently of autonomic input [14,15,16,17,18]. In contrast, metoprolol, a beta-adrenergic blocker, reduces heart rate indirectly by attenuating sympathetic stimulation via beta-receptor inhibition [30]. Consequently, in the context of post-transplant autonomic denervation, the effectiveness of beta-blockers is inherently limited [2,6,7].

### 4.2. Long-Term Results of Side Effects, Blood Pressure, and QT/QTc Interval

Since an elevated resting heart rate after HTX has been associated with reduced post-transplant survival [6,7,12], effective management of sinus tachycardia is essential. Ivabradine is a highly specific and selective inhibitor of the If current, also referred to as the pacemaker current, which is mediated by HCN channels in pacemaker cells of the sinoatrial node (SAN), the heart’s primary pacemaker [1,2,3,4,5,6,7,14,15,16,17,18]. Through this mechanism, ivabradine effectively reduces the resting heart rate without exerting significant effects on other parameters of cardiac function. Despite evidence supporting the safety and efficacy of ivabradine in achieving heart rate reduction in patients after HTX, its application in this patient population remains off-label and necessitates vigilant clinical monitoring [1,2,3,4,5,6,7]. Moreover, its use may be associated with extracardiac adverse effects, such as luminous visual disturbances (phosphenes), which are attributed to the inhibition of similar channels in the retina. These visual phenomena typically emerge approximately 40 days after initiation of therapy, are transient in nature, and generally do not interfere with daily activities [1,2,3,4,5,6,7,14]. In contrast, metoprolol is a non-selective inhibitor of pacemaker activity, which can lead to a range of cardiac and systemic side effects, including decreased contractility, atrioventricular block, low blood pressure, bronchospasm, fatigue, depression, and sexual dysfunction [1,2,3,4,5,6,7,14].

In our study, only three HTX recipients with ivabradine (5.6%) reported transient experiences of phosphenes, while patients with ivabradine had a significantly lower percentage of symptomatic bradycardia with heart rates < 60 bpm (*p* = 0.013), intermittent dizziness (*p* = 0.017), and fatigue (*p* = 0.025) in comparison to patients with metoprolol after HTX. Our findings are consistent with those of Boeken and colleagues [28] as well as with those of Lage-Gallé and colleagues [31] who both reported no substantial adverse effects associated with ivabradine use in HTX recipients [28,31].

With respect to blood pressure, HTX recipients with ivabradine or metoprolol exhibited comparable systolic and diastolic values at baseline after HTX. In the ivabradine group, systolic and diastolic blood pressure remained stable over time, with no significant differences observed between the two groups at both the 5-year and 10-year follow-up after HTX. Furthermore, we observed no significant difference between groups regarding the use of antihypertensive agents. These results align with previous studies reporting no significant impact of ivabradine on systolic or diastolic blood pressure [1,2,3,4,5,6,7,14].

Ivabradine‘s heart rate-lowering properties can lead to bradycardia and QT interval prolongation as the QT interval is inherently rate-dependent [19,20,21,22]. However, from a clinical point of view, the QTc interval is more relevant for assessing the risk of cardiac arrhythmias. In our study, no significant differences were observed between the ivabradine and metoprolol groups in either QT or QTc intervals at baseline after HTX. At 5-year and 10-year follow-up after HTX, patients with ivabradine as well as with metoprolol exhibited a statistically significant prolongation of the QT interval compared to baseline, which corresponds with the observed reduction in heart rate. However, in both groups, QTc intervals remained stable over time, with no significant changes detected at 5-year and 10-year follow-up after HTX compared to baseline. Moreover, there were no statistically or clinically meaningful differences in QT or QTc intervals between the two groups throughout the study period. Additionally, there was no significant difference between both groups regarding the use of tacrolimus (*p* = 0.771), a known QT-prolonging immunosuppressant [23,32,33].

### 4.3. Long-Term Survival After Heart Transplantation

Regulation of resting heart rate is of critical importance in HTX recipients as elevated heart rates have been associated with increased post-transplant mortality [6,7,12]. Ivabradine has demonstrated both safety and efficacy in managing elevated heart rates in this patient population [1,2,3,4,5,6,7]. However, existing evidence is largely confined to short- and medium-term outcomes, with long-term data currently lacking [1,2,3,4,5,6,7,28,29,31]. In the present study, we observed a significantly improved 10-year post-transplant survival in HTX recipients treated with ivabradine (*p* = 0.003), along with a markedly lower percentage of graft failure-related mortality (1.9% vs. 21.4%, *p* = 0.001). Comprehensive analyses of demographics and post-transplant medications, including immunosuppressive drug therapy, revealed no statistically significant differences between groups that could account for the observed survival benefit. Furthermore, multivariate analysis confirmed a significantly reduced risk of mortality within ten years after HTX for patients receiving ivabradine (HR: 0.374, 95% CI: 0.182–0.770, *p* = 0.008), suggesting a favorable impact on long-term survival, extending previous observations of improved short- and mid-term outcomes in HTX recipients [6,7].

In contrast, Dos Santos and colleagues [29] reported no statistically significant reduction in all-cause mortality at 3-year follow-up after HTX in patients treated with ivabradine (*p* = 0.13). However, the authors acknowledged that their study was not powered to detect differences in mortality, noting that a substantially larger sample size with extended follow-up would be needed [29].

The underlying mechanisms contributing to this improved survival in HTX recipients with ivabradine may extend beyond heart rate control. Ivabradine has been associated with enhanced endothelial function, increased sarcoplasmic reticulum calcium uptake, reduced expression of pro-inflammatory cytokines (including tumor necrosis factor alpha), decreased oxidative stress, normalization of mRNA expression across multiple signaling pathways, and reductions in cardiomyocyte apoptosis and hypertrophy [34,35,36]. In addition to these pleiotropic effects, ivabradine-induced heart rate reduction may also contribute to improved long-term post-transplant survival through favorable alterations in echocardiographic parameters, such as LV mass and diastolic function [6,7].

### 4.4. Long-Term Effects of Heart Rate Control After Heart Transplantation

Several mechanisms are likely involved in the cardioprotective effects of ivabradine following HTX [1,2,3,4,5,6,7,37,38,39]. Its primary action—heart rate reduction—prolongs diastolic time, thereby enhancing coronary perfusion, ventricular filling, and diastolic function [34,35,36,37,38,39]. Improved diastolic performance may, in part, be mediated by increased sarcoplasmic reticulum calcium uptake and enhanced SERCA (sarcoplasmic/endoplasmic reticulum calcium ATPase) activity [36]. In the present study, ivabradine-treated HTX recipients exhibited significantly higher E/A ratios and lower E/e′ ratios, consistent with prior reports [36,37].

Beyond its effects on diastolic function, ivabradine has been shown to influence systolic function and myocardial structure through modulation of cardiomyocytes and the extracellular matrix [38,39]. In our cohort, HTX recipients receiving ivabradine had a significantly higher LVEF and a marked reduction in LV mass and the LV mass index toward normative values. These findings suggest that ivabradine may attenuate adverse cardiac remodeling and cardiomyocyte hypertrophy, thereby improving LV function through reduced hypoxia and myocardial oxygen demand [36].

Each heartbeat imposes energetic stress on cardiac metabolism and mechanical stress on the endothelium [35,40]. Elevated heart rates therefore amplify pulsatile wall stress, potentially leading to endothelial dysfunction, inflammation, structural degradation, and microvascular coronary disease [35,36,37,38]. Notably, ivabradine has been reported to stimulate angiogenesis and enhance microvascular perfusion with long-term use [34,35,36,37,38,39]. Although our study found no significant differences between HTX recipients with ivabradine or metoprolol in coronary artery disease, coronary stenting, or high-sensitivity cardiac troponin T, patients treated with ivabradine had a significantly lower LVEDP and NT-proBNP level—findings that may reflect improved microvascular integrity and changes in cardiac metabolism.

Improvement in microvascular perfusion, reduction in myocardial oxygen demand and oxidative stress, as well as vascular protective effects are all clinically relevant, given the fact that approximately one out of three HTX recipients develops cardiac allograft vasculopathy within five years after HTX due to coronary inflammation, endothelial dysfunction, and fibroproliferative remodeling [41].

In summary, our findings suggest that ivabradine not only provides effective heart rate control after HTX but also exerts beneficial effects at multiple levels—enhancing systolic and diastolic function, mitigating hypertrophic remodeling, and supporting coronary microvascular health through angiogenic and anti-oxidative mechanisms [34,35,36,37,38,39,40,41]. Importantly, HTX recipients with ivabradine rapidly achieved a durable resting heart rate ≤ 80 bpm and sustained a resting heart rate ≤ 80 bpm over the entire 10-year study period. This is of great clinical importance as non-transplant participants with a resting heart rate > 80 bpm had a greater risk for both cardiovascular disease and all-cause mortality in a large epidemiological study [42]. Based upon our findings, resting heart rates > 80 bpm in HTX recipients should be addressed and treated with ivabradine, if possible, to achieve an adequate long-term heart rate control after HTX with a resting heart rate ≤ 80 bpm. However, further investigation—ideally through large, multicenter randomized controlled trials—is warranted to explore these findings in greater detail and to validate the long-term benefits of ivabradine in the post-transplant setting.

### 4.5. Study Limitations

Our results were based on an observational retrospective single-center study with 110 adult patients receiving HTX at Heidelberg Heart Center between 2006 and 2015. Hereof, 54 HTX recipients had ivabradine, and 56 HTX recipients had metoprolol. Though this participant number seems rather small at first glance, to our knowledge, this is the largest study analyzing long-term effects (10-year results) of heart rate control with ivabradine or metoprolol in HTX recipients. It therefore provides important and clinically needed data to the limited available literature in this field [1,2,3,4,5,6,7].

Given the known limitations of the study design used, our findings should be interpreted with caution as the non-randomized study design may be subject to selection bias and unmeasured confounders. Nevertheless, we were able to use highly detailed data of 110 HTX recipients, as our patients received standardized treatment and follow-up, reducing the likelihood of selection bias and potential confounders [6,7,24,25,26].

The Devereux formula was used to calculate LV mass, which carries the limitation of a two-dimensional assessment. Assessment of echocardiographic features is generally subject to the examiner‘s experience. Therefore, echocardiographic video files were independently assessed by two cardiologists [6,7].

We did not perform a randomization of HTX recipients regarding the use of ivabradine or metoprolol as individual physician practice and patient preference influenced the prescription reflecting real-world data. However, we could not detect significant differences between groups in terms of demographics or concurrent drugs reducing the likelihood of selection bias [6,7,24,25,26].

We also did not perform routine exercise-based testing such as exercise ECG, treadmill exercise ECG, or a 6 min walk test in HTX recipients at Heidelberg Heart Center but rather performed stress cardiac MRI or cardiac catheterization with biopsy in cases of clinical deterioration, changes in LVEF, or suspicion of relevant myocardial ischemia. We therefore had to rely on a resting 12-lead ECG and 24 h Holter monitor data, which were routinely performed [6,7,24,25,26].

Regarding the use of ivabradine or metoprolol over a period of ten years, temporary changes in medications cannot be ruled out completely, as routine follow-up visits at Heidelberg Heart Center were reduced to once or twice annually, five years after HTX. However, HTX recipients, in general, have a very high rate of medication adherence as this is crucial for their survival. In addition, patients were routinely asked about their medication adherence at each follow-up and change in medications was standardly performed only after consultation [6,7,24,25,26].

Finally, our results should be considered hypothesis-generating, especially in terms of post-transplant survival, as multiple factors may affect post-transplant survival. It is further unknown whether our findings are attributed to differences between ivabradine and metoprolol or ivabradine and beta blockers in general. Therefore, to confirm our findings, further research is needed, ideally through large, prospective randomized controlled multicenter trials to investigate the long-term effects of heart rate control with ivabradine or metoprolol in patients after HTX [6,7,24,25,26].

## 5. Conclusions

As sinus tachycardia after HTX due to cardiac graft denervation is associated with reduced post-transplant survival, we performed an observational retrospective single-center study analyzing the ten-year post-transplant results of 54 HTX recipients with ivabradine and 56 HTX recipients with metoprolol. Noteworthy, HTX recipients were neither preselected nor randomized for treatment with ivabradine versus metoprolol to manage post-transplant heart rate. Individual physician practice and patient preference influenced the prescription of either drug, reflecting real-world data [6,7]. At 10-year follow-up, HTX recipients with ivabradine showed a significantly lower average heart rate compared to baseline (*p* < 0.001) and to metoprolol (*p* < 0.001) as well as a significantly lower NT-proBNP level compared to baseline (*p* < 0.001) and to metoprolol succinate (*p* = 0.005). Moreover, HTX recipients with ivabradine had a significantly lower overall mortality (20.4% versus 46.4%, *p* = 0.004) and mortality due to graft failure (1.9% versus 21.4%, *p* = 0.001) in comparison to HTX recipients with metoprolol at 10-year follow-up. In addition, multivariate analysis showed a significantly decreased risk of death within ten years after HTX in patients with post-transplant use of ivabradine (HR 0.374, CI 0.182–0.770; *p* = 0.008). In summary, based upon our findings, ivabradine appears to be a safe, specific, and selective long-term treatment of sinus tachycardia in HTX recipients, which was associated with a significantly better heart rate reduction, a lower NT-proBNP level, and a superior 10-year survival after HTX.

## Figures and Tables

**Table 1:** Demographic and clinical characteristics at baseline.

Parameter	All Patients (*n* = 110)	Ivabradine (*n* = 54)	Metoprolol (*n* = 56)	Difference	95% CI	*p*-Value
Recipient data						
Age (years), mean ± SD	51.3 ± 10.8	49.9 ± 11.4	52.7 ± 10.2	2.8	−1.2–6.8	0.165
Male sex, *n* (%)	82 (74.5%)	40 (74.1%)	42 (75.0%)	0.9%	−15.4–17.2%	0.911
BMI (kg/m^2^), mean ± SD	25.3 ± 4.7	24.8 ± 4.9	25.8 ± 4.4	1.0	−0.7–2.7	0.226
Arterial hypertension, *n* (%)	65 (59.1%)	28 (51.9%)	37 (66.1%)	14.2%	−4.0–32.4%	0.129
Dyslipidemia, *n* (%)	66 (60.0%)	32 (59.2%)	34 (60.7%)	1.5%	−16.8–19.8%	0.876
Diabetes mellitus, *n* (%)	36 (32.7%)	16 (29.6%)	20 (35.7%)	6.1%	−11.4–23.6%	0.497
Peripheral artery disease, *n* (%)	11 (10.0%)	3 (5.6%)	8 (14.3%)	8.7%	−2.3–19.7%	0.127
COPD, *n* (%)	26 (23.6%)	9 (16.7%)	17 (30.4%)	13.7%	−1.9–29.3%	0.091
History of smoking, *n* (%)	65 (59.1%)	29 (53.7%)	36 (64.3%)	10.6%	−7.7–28.9%	0.259
Chronic kidney disease ^, *n* (%)	59 (53.6%)	27 (50.0%)	32 (57.1%)	7.1%	−11.5–25.7%	0.453
eGFR (ml/min/1.73 m^2^), mean ± SD	59.7 ± 22.6	61.6 ± 25.0	57.8 ± 20.1	3.8	−4.7–12.3	0.391
Previous open-heart surgery						
Overall open-heart surgery, *n* (%)	21 (19.1%)	11 (20.4%)	10 (17.9%)	2.5%	−12.2–17.2%	0.737
CABG surgery, *n* (%)	8 (7.3%)	3 (5.6%)	5 (8.9%)	3.3%	−6.4–13.0%	0.496
Other surgery °, *n* (%)	8 (7.3%)	4 (7.4%)	4 (7.1%)	0.3%	−9.4–10.0%	0.957
VAD surgery, *n* (%)	8 (7.3%)	5 (9.3%)	3 (5.4%)	3.9%	−5.9–13.7%	0.431
Principal diagnosis for HTX						
Ischemic CMP, *n* (%)	38 (34.5%)	15 (27.8%)	23 (41.1%)	13.3%	−4.3–30.9%	0.143
Non-ischemic CMP, *n* (%)	53 (48.2%)	30 (55.5%)	23 (41.1%)	14.4%	−4.1–32.9%	0.129
Valvular heart disease, *n* (%)	3 (2.7%)	2 (3.7%)	1 (1.8%)	1.9%	−4.2–8.0%	0.537
Cardiac amyloidosis, *n* (%)	16 (14.6%)	7 (13.0%)	9 (16.0%)	3.0%	−10.1–16.1%	0.644
Donor data						
Age (years), mean ± SD	45.2 ± 13.5	42.9 ± 14.6	47.4 ± 12.1	4.5	−0.5–9.5	0.083
Male sex, *n* (%)	31 (28.2%)	13 (24.1%)	18 (32.1%)	8.0%	−8.7–24.7%	0.347
BMI (kg/m^2^), mean ± SD	25.3 ± 5.2	24.5 ± 5.2	26.1 ± 5.1	1.6	−0.3–3.5	0.099
Transplant sex mismatch						
Mismatch, *n* (%)	58 (52.7%)	32 (59.2%)	26 (46.4%)	12.8%	−5.7–31.3%	0.178
Donor (m) to recipient (f), *n* (%)	3 (2.7%)	2 (3.7%)	1 (1.8%)	1.9%	−4.2–8.0%	0.537
Donor (f) to recipient (m), *n* (%)	55 (50.7%)	30 (55.5%)	25 (44.6%)	10.9%	−7.7–29.5%	0.252
Perioperative data						
Ischemic time (min), mean ± SD	262.4 ± 56.6	268.9 ± 52.7	256.1 ± 59.8	12.8	−8.3–33.9	0.236
Biatrial anastomosis, *n* (%)	1 (0.9%)	0 (0.0%)	1 (1.8%)	1.8%	−1.7–5.3%	0.324
Bicaval anastomosis, *n* (%)	47 (42.7%)	27 (50.0%)	20 (35.7%)	14.3%	−4.0–32.6%	0.130
Total orthotopic anastomosis, *n* (%)	62 (56.4%)	27 (50.0%)	35 (62.5%)	12.5%	−5.9–30.9%	0.186
LOS (days), mean ± SD	45.4 ± 19.4	44.8 ± 20.0	46.1 ± 18.9	1.3	−6.0–8.6	0.721

BMI = body mass index; CABG = coronary artery bypass graft; CI = confidence interval; CMP = cardiomyopathy; COPD = chronic obstructive pulmonary disease; f = female; eGFR = estimated glomerular filtration rate; HTX = heart transplantation; LOS = length of initial hospital stay; m = male; *n* = number; SD = standard deviation; VAD = ventricular assist device; ^ = eGFR < 60 mL/min/1.73 m^2^; and ° = congenital, valvular, or ventricular surgery.

**Table 2:** Post-transplant medications at baseline.

Parameter	All Patients (*n* = 110)	Ivabradine (*n* = 54)	Metoprolol (*n* = 56)	Difference	95% CI	*p*-Value
Immunosuppressive drug therapy						
Cyclosporine A, *n* (%)	9 (8.2%)	4 (7.4%)	5 (8.9%)	1.5%	−8.7–11.7%	0.771
Tacrolimus, *n* (%)	101 (91.8%)	50 (92.6%)	51 (91.1%)	1.5%	−8.7–11.7%	0.771
Azathioprine, *n* (%)	0 (0.0%)	0 (0.0%)	0 (0.0%)	0.0%	n. a.	n. a.
Mycophenolic acid, *n* (%)	110 (100.0%)	54 (100.0%)	56 (100.0%)	0.0%	n. a.	n. a.
Steroids, *n* (%)	110 (100.0%)	54 (100.0%)	56 (100.0%)	0.0%	n. a.	n. a.
Concomitant medications						
ASA, *n* (%)	21 (19.1%)	8 (14.8%)	13 (23.2%)	8.4%	−6.2–23.0%	0.262
Amiodarone, *n* (%)	0 (0.0%)	0 (0.0%)	0 (0.0%)	0.0%	n. a.	n. a.
Digitalis, *n* (%)	0 (0.0%)	0 (0.0%)	0 (0.0%)	0.0%	n. a.	n. a.
Beta blocker, *n* (%)	56 (50.9%)	0 (0.0%)	56 (100.0%)	100.0%	n. a.	<0.001 *
Ivabradine, *n* (%)	54 (49.1%)	54 (100.0%)	0 (0.0%)	100.0%	n. a.	<0.001 *
Calcium channel blocker ^, *n* (%)	25 (22.7%)	12 (22.2%)	13 (23.2%)	1.0%	−14.7–16.7%	0.901
ACE inhibitor/ARB, *n* (%)	46 (41.8%)	23 (42.6%)	23 (41.1%)	1.5%	−16.9–19.9%	0.872
Diuretic, *n* (%)	110 (100.0%)	54 (100.0%)	56 (100.0%)	0.0%	n. a.	n. a.
Statin, *n* (%)	80 (72.7%)	37 (68.5%)	43 (76.8%)	8.3%	−8.3–24.9%	0.330
Gastric protection drug ^†^, *n* (%)	110 (100.0%)	54 (100.0%)	56 (100.0%)	0.0%	n. a.	n. a.

ACE inhibitor = angiotensin-converting-enzyme inhibitor; ARB = angiotensin II receptor blocker; ASA = acetylsalicylic acid; CI = confidence interval; *n* = number; n. a. = not applicable; ^†^ = gastric protection drug defined as proton pump inhibitor (PPI) or histamine receptor (H_2_) blocker; ^ = dihydropyridine calcium channel blocker; and * = statistically significant (*p* < 0.050).

**Table 3:** Resting ECG, 24 h-Holter monitor, QT/QTc intervals, and blood pressure values.

Parameter	All Patients (*n* = 110)	Ivabradine (*n* = 54)	Metoprolol (*n* = 56)	Difference	95% CI	*p*-Value
Average heart rate (bpm) (resting ECG), mean ± SD						
Baseline after HTX	87.8 ± 8.6	88.8 ± 7.6	86.9 ± 9.5	1.9	−1.3–5.1	0.257
At 5-year FU after HTX	76.7 ± 10.2	73.4 ± 9.0	80.7 ± 10.3	7.3	3.2–11.4	<0.001 *
At 10-year FU after HTX	75.7 ± 9.0	72.7 ± 8.5	80.1 ± 8.1	7.4	3.6–11.2	<0.001 *
*p*-value: baseline vs. 5-year FU	<0.001 *	<0.001 *	0.004 *			
*p*-value: baseline vs. 10-year FU	<0.001 *	<0.001 *	0.001 *			
Average heart rate (bpm) (24 h-Holter monitor), mean ± SD						
Baseline after HTX	85.9 ± 9.4	86.2 ± 9.8	85.6 ± 9.0	0.6	−2.9–4.1	0.756
At 5-year FU after HTX	75.9 ± 8.4	72.5 ± 7.1	79.9 ± 8.1	7.4	4.1–10.7	<0.001 *
At 10-year FU after HTX	74.2 ± 8.6	70.9 ± 7.0	79.1 ± 8.4	8.2	4.5–11.9	<0.001 *
*p*-value: baseline vs. 5-year FU	<0.001 *	<0.001 *	0.002 *			
*p*-value: baseline vs. 10-year FU	<0.001 *	<0.001 *	0.001 *			
QT interval (ms), mean ± SD						
Baseline after HTX	367.5 ± 21.2	365.5 ± 21.1	369.4 ± 21.2	3.9	−4.0–11.8	0.337
At 5-year FU after HTX	388.0 ± 23.8	391.9 ± 23.4	383.5 ± 23.8	8.4	−1.6–18.4	0.105
At 10-year FU after HTX	391.6 ± 24.3	392.8 ± 26.4	389.9 ± 21.3	2.9	−8.1–13.9	0.603
*p*-value: baseline vs. 5-year FU	<0.001 *	<0.001 *	0.004 *			
*p*-value: baseline vs. 10-year FU	<0.001 *	<0.001 *	<0.001 *			
QTc interval (ms), mean ± SD						
Baseline after HTX	416.0 ± 19.4	415.2 ± 21.2	416.8 ± 17.6	1.6	−5.7–8.9	0.672
At 5-year FU after HTX	419.4 ± 16.4	417.3 ± 14.9	422.0 ± 18.0	4.7	−2.4–11.8	0.199
At 10-year FU after HTX	420.3 ± 16.9	419.0 ± 17.8	422.2 ± 15.5	3.2	−4.5–10.9	0.423
*p*-value: baseline vs. 5-year FU	0.182	0.560	0.165			
*p*-value: baseline vs. 10-year FU	0.115	0.341	0.148			
Systolic BP (mmHg), mean ± SD						
Baseline after HTX	125.8 ± 15.4	126.1 ± 13.9	125.4 ± 16.8	0.7	−5.1–6.5	0.821
At 5-year FU after HTX	125.5 ± 11.7	125.2 ± 13.3	125.9 ± 9.5	0.7	−4.1–5.5	0.782
At 10-year FU after HTX	124.6 ± 12.2	124.5 ± 12.8	124.7 ± 11.5	0.2	−5.4–5.8	0.963
*p*-value: baseline vs. 5-year FU	0.897	0.741	0.869			
*p*-value: baseline vs. 10-year FU	0.564	0.562	0.801			
Diastolic BP (mmHg), mean ± SD						
Baseline after HTX	77.3 ± 9.1	78.0 ± 9.3	76.7 ± 9.0	1.3	−2.1–4.7	0.469
At 5-year FU after HTX	76.9 ± 8.9	76.7 ± 7.5	77.2 ± 8.8	0.5	−3.0–4.0	0.790
At 10-year FU after HTX	76.2 ± 8.7	76.2 ± 9.7	76.3 ± 7.3	0.1	−3.8–4.0	0.932
*p*-value: baseline vs. 5-year FU	0.746	0.454	0.794			
*p*-value: baseline vs. 10-year FU	0.419	0.357	0.840			

BP = blood pressure; BPM = beats per minute; CI = confidence interval; FU = follow-up; HTX = heart transplantation; ms = milliseconds; *n* = number; SD = standard deviation; vs. = versus; * = statistically significant (*p* < 0.050).

**Table 4:** Causes of death after HTX.

**(a) Within 5 Years After HTX.**						
**Parameter**	**All Patients** ** (*n* = 110)**	**Ivabradine** ** (*n* = 54)**	**Metoprolol** ** (*n* = 56)**	**Difference**	**95% CI**	***p*-Value**
Graft failure, *n* (%)	9 (8.2%)	0 (0.0%)	9 (16.0%)	16.0%	6.4–25.6%	0.002 *
Acute rejection, *n* (%)	2 (1.8%)	1 (1.9%)	1 (1.8%)	0.1%	−4.9–5.1%	0.979
Infection/sepsis, *n* (%)	11 (10.0%)	5 (9.2%)	6 (10.7%)	1.5%	−9.7–12.7%	0.799
Malignancy, *n* (%)	1 (0.9%)	1 (1.9%)	0 (0.0%)	1.9%	−1.7–5.5%	0.306
Thromboembolic event/bleeding, *n* (%)	1 (0.9%)	0 (0.0%)	1 (1.8%)	1.8%	−1.7–5.3%	0.324
All causes, *n* (%)	24 (21.8%)	7 (13.0%)	17 (30.3%)	17.3%	2.3–32.3%	0.027 *
**(b) Within 10 Years After HTX.**						
**Parameter**	**All Patients** ** (*n* = 110)**	**Ivabradine** ** (*n* = 54)**	**Metoprolol** ** (*n* = 56)**	**Difference**	**95% CI**	***p*-Value**
Graft failure, *n* (%)	13 (11.8%)	1 (1.9%)	12 (21.4%)	19.5%	8.2–30.8%	0.001 *
Acute rejection, *n* (%)	3 (2.7%)	1 (1.9%)	2 (3.6%)	1.7%	−4.4–7.8%	0.580
Infection/sepsis, *n* (%)	15 (13.6%)	7 (13.0%)	8 (14.3%)	1.3%	−11.5–14.1%	0.840
Malignancy, *n* (%)	4 (3.6%)	2 (3.7%)	2 (3.6%)	0.1%	−6.9–7.1%	0.970
Thromboembolic event/bleeding, *n* (%)	2 (1.8%)	0 (0.0%)	2 (3.6%)	3.6%	−1.3–8.5%	0.161
All causes, *n* (%)	37 (33.6%)	11 (20.4%)	26 (46.4%)	26.0%	9.1–42.9%	0.004 *

CI = confidence interval; HTX = heart transplantation; *n* = number; and* = statistically significant (*p* < 0.050).

**Table 5:** Multivariate analysis for mortality after HTX.

**(a) Within 5 Years After HTX.**			
**Parameter**	**Hazard Ratio**	**95% CI**	***p*-Value**
Recipient age (years)	0.989	0.947–1.034	0.631
Recipient BMI (kg/m^2^)	1.050	0.961–1.147	0.278
Recipient eGFR (mL/min/1.73 m^2^)	1.003	0.984–1.022	0.791
Donor age (years)	0.996	0.962–1.031	0.805
Donor BMI (kg/m^2^)	1.072	0.991–1.159	0.082
Transplant sex mismatch (in total)	0.943	0.412–2.161	0.890
Total ischemic time (min)	1.001	0.994–1.008	0.841
Administration of ivabradine after HTX (in total)	0.388	0.159–0.949	0.038 *
**(b) Within 10 Years After HTX.**			
**Parameter**	**Hazard Ratio**	**95% CI**	***p*-Value**
Recipient age (years)	0.982	0.949–1.017	0.314
Recipient BMI (kg/m^2^)	1.036	0.963–1.115	0.341
Recipient eGFR (mL/min/1.73 m^2^)	1.003	0.987–1.020	0.687
Donor age (years)	1.008	0.980–1.037	0.566
Donor BMI (kg/m^2^)	1.038	0.971–1.110	0.278
Transplant sex mismatch (in total)	0.940	0.480–1.839	0.856
Total ischemic time (min)	1.001	0.995–1.006	0.769
Administration of ivabradine after HTX (in total)	0.374	0.182–0.770	0.008 *

BMI = body mass index; CI = confidence interval; eGFR = estimated glomerular filtration rate; HTX = heart transplantation; and * = statistically significant (*p* < 0.050).

**Table 6:** Echocardiographic features after HTX.

Parameter	All Patients (*n* = 110)	Ivabradine (*n* = 54)	Metoprolol (*n* = 56)	Difference	95% CI	*p*-Value
LV mass (g), mean ± SD						
Baseline after HTX	178.6 ± 40.4	178.5 ± 41.9	178.6 ± 39.3	0.1	−15.0–15.2	0.993
At 5-year FU after HTX	165.0 ± 28.6	153.7 ± 23.7	178.2 ± 28.4	24.5	13.4–35.6	<0.001 *
At 10-year FU after HTX	165.8 ± 34.6	155.9 ± 32.4	179.9 ± 33.2	24.0	8.6–39.4	0.003 *
*p*-value: baseline vs. 5-year FU	0.006 *	<0.001 *	0.949			
*p*-value: baseline vs. 10-year FU	0.023 *	0.004 *	0.874			
LV mass index (g/m^2^), mean ± SD						
Baseline after HTX	94.3 ± 20.9	96.1 ± 21.4	92.5 ± 20.4	3.6	−4.2–11.4	0.363
At 5-year FU after HTX	87.2 ± 17.1	81.7 ± 12.9	93.8 ± 19.2	12.1	5.1–19.1	0.001 *
At 10-year FU after HTX	86.8 ± 17.1	83.1 ± 15.8	92.0 ± 17.7	8.9	1.0–16.8	0.031 *
*p*-value: baseline vs. 5-year FU	0.010 *	<0.001 *	0.756			
*p*-value: baseline vs. 10-year FU	0.009 *	0.001 *	0.916			
LVEF (%), mean ± SD						
Baseline after HTX	62.1 ± 4.2	61.6 ± 4.2	62.6 ± 4.2	1.0	−0.6–2.6	0.198
At 5-year FU after HTX	59.1 ± 6.2	61.5 ± 6.1	56.3 ± 5.0	5.2	2.9–7.5	<0.001 *
At 10-year FU after HTX	58.2 ± 5.8	60.7 ± 4.5	54.6 ± 5.8	6.1	3.7–8.5	<0.001 *
*p*-value: baseline vs. 5-year FU	<0.001 *	0.954	<0.001 *			
*p*-value: baseline vs. 10-year FU	<0.001 *	0.314	<0.001 *			
MAPSE (mm), mean ± SD						
Baseline after HTX	17.7 ± 1.7	17.4 ± 1.8	17.9 ± 1.6	0.5	−0.2–1.2	0.168
At 5-year FU after HTX	16.6 ± 2.8	17.5 ± 2.5	15.4 ± 2.8	2.1	1.0–3.2	<0.001 *
At 10-year FU after HTX	16.3 ± 2.5	17.1 ± 2.4	15.1 ± 2.2	2.0	0.9–3.1	<0.001 *
*p*-value: baseline vs. 5-year FU	0.002 *	0.808	<0.001 *			
*p*-value: baseline vs. 10-year FU	<0.001 *	0.515	<0.001 *			
E/A ratio, mean ± SD						
Baseline after HTX	1.5 ± 0.4	1.5 ± 0.3	1.6 ± 0.4	0.1	−0.1–0.3	0.817
At 5-year FU after HTX	1.5 ± 0.5	1.6 ± 0.4	1.3 ± 0.5	0.3	0.1–0.5	<0.001 *
At 10-year FU after HTX	1.5 ± 0.4	1.6 ± 0.4	1.3 ± 0.4	0.3	0.1–0.5	<0.001 *
*p*-value: baseline vs. 5-year FU	0.236	0.175	0.003 *			
*p*-value: baseline vs. 10-year FU	0.473	0.169	0.005 *			
E/e′ ratio, mean ± SD						
Baseline after HTX	7.5 ± 2.7	7.4 ± 2.6	7.6 ± 2.8	0.2	−0.8–1.2	0.805
At 5-year FU after HTX	8.4 ± 3.4	7.2 ± 2.6	9.9 ± 3.8	2.7	1.3–4.1	<0.001 *
At 10-year FU after HTX	8.4 ± 2.7	7.4 ± 1.8	9.8 ± 3.1	2.4	1.2–3.6	<0.001 *
*p*-value: baseline vs. 5-year FU	0.040 *	0.683	0.002 *			
*p*-value: baseline vs. 10-year FU	0.027 *	0.987	0.002 *			
DT-E (ms), mean ± SD						
Baseline after HTX	211.1 ± 24.0	211.8 ± 22.5	210.4 ± 25.6	1.4	−7.6–10.4	0.766
At 5-year FU after HTX	192.2 ± 25.9	180.6 ± 21.1	205.8 ± 24.6	25.2	15.5–34.9	<0.001 *
At 10-year FU after HTX	190.2 ± 27.8	181.9 ± 28.8	202.2 ± 21.5	20.3	8.8–31.8	<0.001 *
*p*-value: baseline vs. 5-year FU	<0.001 *	<0.001 *	0.369			
*p*-value: baseline vs. 10-year FU	<0.001 *	<0.001 *	0.117			
LA diameter (mm), mean ± SD						
Baseline after HTX	38.8 ± 5.5	38.4 ± 5.8	39.1 ± 5.3	0.7	−1.4–2.8	0.458
At 5-year FU after HTX	40.6 ± 5.0	39.3 ± 4.9	42.2 ± 4.6	2.9	0.9–4.9	0.006 *
At 10-year FU after HTX	41.4 ± 4.5	40.2 ± 4.5	43.1 ± 3.9	2.9	1.0–4.8	0.005 *
*p*-value: baseline vs. 5-year FU	0.015 *	0.389	0.004 *			
*p*-value: baseline vs. 10-year FU	<0.001 *	0.076	<0.001 *			
Systolic PAP (mmHg), mean ± SD						
Baseline after HTX	30.6 ± 7.5	30.5 ± 7.5	30.6 ± 7.7	0.1	−2.7–2.9	0.901
At 5-year FU after HTX	27.9 ± 7.0	25.8 ± 7.1	30.4 ± 6.0	4.6	1.8–7.4	0.002 *
At 10-year FU after HTX	28.1 ± 6.4	26.5 ± 5.4	30.5 ± 7.1	4.0	1.0–7.0	0.012 *
*p*-value: baseline vs. 5-year FU	0.011 *	0.002 *	0.835			
*p*-value: baseline vs. 10-year FU	0.020 *	0.003 *	0.915			

CI = confidence interval; DT-E = deceleration time (DT) of the early diastolic mitral inflow peak (E); E/A = early diastolic mitral inflow peak velocity (E) to late diastolic mitral inflow peak velocity (A) ratio; E/e′ = early diastolic mitral inflow peak velocity (E) to early diastolic mitral annular velocity (e′) ratio; FU = follow-up; HTX = heart transplantation; LA = left atrial; LV = left ventricular; LVEF = left ventricular ejection fraction; MAPSE = mitral annular plane systolic excursion; *n* = number; PAP = pulmonary artery pressure; SD = standard deviation; vs. = versus; and * = statistically significant (*p* < 0.050).

**Table 7:** Cardiac catheterization data and cardiac biomarkers after HTX.

Parameter	All Patients (*n* = 110)	Ivabradine (*n* = 54)	Metoprolol (*n* = 56)	Difference	95% CI	*p*-Value
CAD (stenosis ≥ 50%), *n* (%)						
Baseline after HTX	5 (4.5%)	3 (5.6%)	2 (3.6%)	2.0%	−5.8–9.8%	0.617
Within 5 years after HTX	27 (24.5%)	14 (25.9%)	13 (23.2%)	2.7%	−13.4–18.8%	0.741
Within 10 years after HTX	42 38.2%)	22 (40.7%)	20 (35.7%)	5.0%	−13.1–23.1%	0.587
*p*-value: baseline vs. within 5 years	<0.001 *	0.004 *	0.002 *			
*p*-value: baseline vs. within 10 years	<0.001 *	<0.001 *	<0.001 *			
Coronary stenting, *n* (%)						
Baseline after HTX	0 (0.0%)	0 (0.0%)	0 (0.0%)	0.0%	n. a.	n. a.
Within 5 years after HTX	12 (10.9%)	6 (11.1%)	6 (10.7%)	0.4%	−11.3–12.1%	0.947
Within 10 years after HTX	27 (24.5%)	15 (27.8%)	12 (21.4%)	6.4%	−9.7–22.5%	0.439
*p*-value: baseline vs. within 5 years	<0.001 *	0.012 *	0.012 *			
*p*-value: baseline vs. within 10 years	<0.001 *	<0.001 *	<0.001 *			
LVEDP (mmHg), mean ± SD						
Baseline after HTX	15.3 ± 2.4	15.4 ± 2.7	15.2 ± 2.0	0.2	−0.7–1.1	0.620
At 5-year FU after HTX	14.3 ± 4.1	12.0 ± 3.7	17.1 ± 2.6	5.1	3.7–6.5	<0.001 *
At 10-year FU after HTX	13.3 ± 4.4	10.4 ± 3.4	16.7 ± 2.5	6.3	5.1–7.5	<0.001*
*p*-value: baseline vs. 5-year FU	0.053	<0.001 *	<0.001 *			
*p*-value: baseline vs. 10-year FU	<0.001 *	<0.001 *	0.002 *			
hs-cTnT (pg/mL), mean ± SD						
Baseline after HTX	167.8 ± 74.4	168.7 ± 68.3	166.8 ± 80.4	1.9	−26.1–29.9	0.892
At 5-year FU after HTX	16.1 ± 11.3	14.6 ± 11.5	17.8 ± 11.0	3.2	−1.5–7.9	0.191
At 10-year FU after HTX	19.5 ± 14.7	18.8 ± 15.0	20.6 ± 14.3	1.8	−5.0–8.6	0.598
*p*-value: baseline vs. 5-year FU	<0.001 *	<0.001 *	<0.001 *			
*p*-value: baseline vs. 10-year FU	<0.001 *	<0.001 *	<0.001 *			
NT-proBNP (pg/mL), mean ± SD						
Baseline after HTX	3853.9 ± 1842.9	3849.7 ± 1960.0	3858.0 ± 1740.3	8.3	−683.8–700.4	0.982
At 5-year FU after HTX	769.5 ± 741.0	555.4 ± 541.9	1021.0 ± 862.8	465.6	156.6–774.6	0.004 *
At 10-year FU after HTX	851.6 ± 843.2	588.4 ± 461.4	1229.0 ± 1098.6	640.6	224.0–1057.2	0.005 *
*p*-value: baseline vs. 5-year FU	<0.001 *	<0.001 *	<0.001 *			
*p*-value: baseline vs. 10-year FU	<0.001 *	<0.001 *	<0.001 *			

CAD = coronary artery disease; CI = confidence interval; FU = follow-up; hs-cTnT = high-sensitivity cardiac troponin T; HTX = heart transplantation; LVEDP = left ventricular end-diastolic pressure; *n* = number; n. a. = not applicable; NT-proBNP = N-terminal prohormone of brain natriuretic peptide; SD = standard deviation; vs. = versus; and * = statistically significant (*p* < 0.050).

## Data Availability

The original contributions presented in this study are included in this article; further inquiries can be directed to the corresponding author.
